# Associations of blood lead, cadmium, and mercury with resistant hypertension among adults in NHANES, 1999–2018

**DOI:** 10.1265/ehpm.23-00151

**Published:** 2023-11-02

**Authors:** Hao Chen, Yunfeng Zou, Xuebing Leng, Feng Huang, Rongjie Huang, Akemi Wijayabahu, Xinguang Chen, Yunan Xu

**Affiliations:** 1 Department of Occupational and Environmental Health, School of Public Health, Guangxi Medical University, Nanning, 530021, China; 2 Department of Microbiology and Immunology, Miller School of Medicine, University of Miami, Miami, FL, US; 3 Department of Cardiology, The First Affiliated Hospital of Guangxi Medical University, Nanning, 530021, China; 4 Guangxi Key Laboratory of Precision Medicine in Cardio-Cerebrovascular Diseases, Nanning, 530021, China; 5 Guangxi Clinical Research Center for Cardio-Cerebrovascular Diseases, Nanning, 530021, China; 6 Department of Epidemiology, University of Florida, Gainesville, Florida, USA; 7 Department of Environmental and Occupational Health, George Washington University, Washington, D.C., USA; 8 Global Health Institute, Xi’an Jiaotong University, Xi’an, 710020, China; 9 Department of Medical Research, The First Affiliated Hospital of Guangxi Medical University, Nanning, 530021, China

**Keywords:** Environmental metal, Resistant hypertension, Lead, Cadmium, NHANES

## Abstract

**Background:**

Resistant hypertension (RHTN), a clinically complex condition with profound health implications, necessitates considerable time and allocation of medical resources for effective management. Unraveling the environmental risk factors associated with RHTN may shed light on future interventional targets aimed at reducing its incidence. Exposure to heavy metal has been linked to an increased risk of hypertension, while the relationship with RHTN remains poorly understood.

**Methods:**

Using the 1999–2018 National Health and Nutrition Examination Survey (NHANES) data, we examined the association of blood lead (Pb), cadmium (Cd), and mercury (Hg) with RHTN using a multinomial logistic regression model. The combined effects of the metals and the contribution of each metal were assessed using a weighted quantile sum (WQS) analysis.

**Results:**

A total of 38281 participants were included in the analysis. Compared with no resistant hypertension (NRHTN), per 1 µg/dL increase in blood Pb concentration, the proportion of RHTN increased by 16% [adjusted odds ratio (aOR), 1.16; 95% confidence interval (CI) 1.01–1.32]. When analyzed by quartiles (Q), the aOR [95% CI] for Pd was 1.30[1.01,1.67] (Q4 vs. Q1); there was a significant dose-response relationship (p < 0.05). Likewise, as a continuous variable, each 1 µg/dL increase in blood Cd level was associated with a 13% increase in the proportion of RHTN (aOR: 1.13; 95%CI: [1.00,1.27]); when analyzed as quartile, aOR [95% CI] for Cd were 1.30[1.01,1.69] (Q3 vs. Q1), and 1.35[1.03,1.75] (Q4 vs. Q1); the dose-response relationship was significant (p < 0.05). WQS analysis showed a significant combined effects of Pb, Cd, and Hg on RHTN, with Pb as the highest weight (0.64), followed by Cd (0.25) and Hg (0.11). Stratified analysis indicated that the associations for the two heavy metals were significant for participants who were male, ⩽60 years old, and with kidney dysfunction.

**Conclusion:**

Findings of this study with national data provide new evidence regarding the role of environmental heavy metal exposure in RHTN. The prevention strategies aimed at reducing heavy metal exposure should particularly focus on Americans who are middle-aged, male, and afflicted with kidney dysfunction.

**Supplementary information:**

The online version contains supplementary material available at https://doi.org/10.1265/ehpm.23-00151.

## Introduction

Hypertension (HTN) is a serious medical condition and a recognized risk factor for cardiovascular and chronic kidney dysfunction (CKD), as well as all-cause mortality [1]. In recent years, resistant hypertension (RHTN) has become a focal point of research. RHTN is defined as the blood pressure (BP) of a hypertensive patient remains elevated above the control goal despite the concurrent use of 3 antihypertensive medications of different classes, including renin-angiotensin system blockers (e.g., angiotensin-converting enzyme [ACE] inhibitor, or angiotensin receptor blocker [ARB]) and diuretics, or BP achieves target values on ≥4 antihypertensive medications [2].

Many adults may suffer from RHTN in the US. Reported studies indicated that approximately 10–30% of the hypertensive patients are RHTN [3, 4]. Compared to non-resistant hypertensive patients (NRHTN), the risk for RHTN patients to develop cardiovascular complications increases by 38%, renal events by 28%, chronic heart failure by 66%, and all-cause mortality by 24%, respectively [5]. Given the significant health burden associated with RHTN, it is imperative to identify potential risk factors linked to this condition, which may provide insights for future interventions aimed at reducing the incidence of RHTN.

Despite the high proportion and excess health risk, the mechanisms underlying RHTN is unclear. BP is regulated by multiple compensatory systems including vascular tone, sodium excretion and plasma volume, and autonomic nervous system [2]. It is likely that all these systems might be involved and controlled by genetic characteristics that result in the pathogenesis of RHTN [2]. The role of renal dysfunctions including renal artery stenosis, primary aldosteronism, and phaeochromocytoma and paraganglioma are also potential risk factors of RHTN [6].

Extensive evidence from the literature has suggested possible links between exposure to environmental metals and elevated risk of cardiovascular diseases, such as congenital heart defects and HTN [7, 8]. For example, elevated blood lead (Pb) levels were associated with higher prevalence of HTN and uncontrolled HTN based on the National Health and Nutrition Examination Survey (NHANES) data from 1999–2016 [9]. A cross-sectional study based on the data from the China National Human Biomonitoring Program reported exposure to a mixture of 11 metals including Pb and arsenic (As) was associated with elevated risk of higher BP [10]. Other environmental metals that have been implicated with higher risk of hypertension include cadmium (Cd), cobalt (Co), mercury (Hg), and magnesium (Mn) [10–14]. An extensive review including 15 studies published between 1998 and 2021 reported that Cd in blood or urine was found to be associated with HTN in all 15 papers, followed by Pb (14 papers), and Hg (11 papers) [11].

In addition to HTN, exposure to environmental metal pollutants may increase the risk of RHTN. However, we only located one study [15] on this issue after an extensive literature search. In the Veterans Affairs Normative Aging Study, Zheutlin and colleagues observed that an interquartile range increase in the tibia Pb level was associated with 19% higher risk of RHTN [15]. The purpose of this study is to examine the relationship between environmental exposure to Pb, Cd and Hg and RHTN based on the NHANES database from 1999–2018. Given that the health effects of metals is varied by sex, age and kidney function [9, 16–20], we hypothesized that exposure to these heavy metals was associated with elevated prevalence of RHTN and the association was not homogeneous but varied by sex, age and CKD.

## Methods

### Study sample

We used data collected by NHANES, a national survey study using the multistage probability sampling of the noninstitutionalized US population. The NHANES conducts surveys in 2-year cycles to assess health and nutritional status among the US population. It is a publicly available dataset without personal identifiable information and has been used by researchers worldwide. Survey protocols were approved by the National Center for Health Statistics Research Ethics Review Board, and written informed consent was obtained from every participant.

**Fig. 1:**
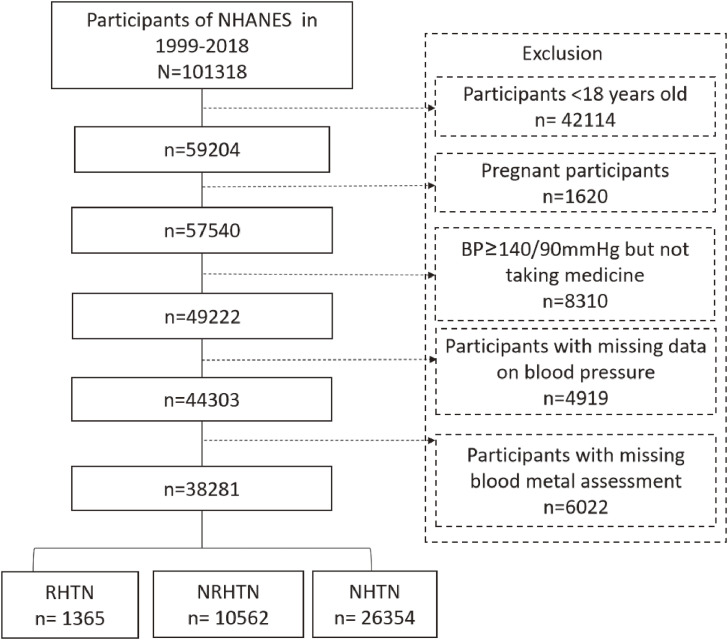
Flow chart of eligible participant selection. BP: blood pressure; RHTN: resistant hypertension; NRHTN: non-resistant hypertension; NHTN: no hypertension

In this study, we used data from 10 consecutive NHANES survey cycles covering the periods 1999–2018. A total of 101,318 participants were included (1999–2000 [n = 9965], 2001–2002 [n = 11039], 2003–2004 [n = 10122], 2005–2006 [n = 10348], 2007–2008 [n = 10149], 2009–2010 [n = 10537], 2011–2012 [n = 9756], 2013–2014 [n = 10175], 2015–2016 [n = 9971], and 2017–2018 [n = 9245]). We excluded individuals who were younger than 18 years old (n = 42114) or pregnant (n = 1620), had systolic blood pressure (SBP) ≥ 140 mmHg and/or diastolic blood pressure (DBP) ≥ 90 mmHg but did not use any antihypertensive medications (n = 8310), no BP measurement (n = 4919), and no blood metal measurement (n = 6022), yielding a final sample of 38281 participants (Fig. 1). Compared to the included individuals, those who had a BP ≥140/90 mmHg but did not use antihypertensive medications tended to be elderly, male, and ethnic minorities. They also had lower levels of education (high school or below) and household income (<$20,000), as well as higher proportion of obesity, smoking and CKD (Table S1). The participants excluded due to the lack of data on BP and metal measurements tended to be female gender and ethnic minorities with annual household income <$20,000, high school or less education, CKD, and diabetes (Table S1).

### Hypertension and resistant hypertension

Three consecutive BP measurements were taken for all individuals following the standard procedure developed by the American Heart Association. A fourth measurement was taken if required. The mean systolic and diastolic blood pressures were calculated using the three or four BP measurements based on whether data of the fourth measurement were available. Participants were classified as HTN if their systolic and/or diastolic BP at or above 140/90 mmHg or reported currently taking antihypertensive medications to the Blood Pressure and Cholesterol questionnaire or Prescription Medications questionnaire. During each interview, the participants were asked to report the medications that they have taken in the past 30 days. When interviewers entered a medication name into the computer, the name was automatically matched to the Multum Lexicon drug database (https://www.cerner.com/solutions/drug-database). The Multum Lexicon encompasses a hierarchical categorization system consisting of either two or three levels, which assigns a therapeutic classification to each drug and each ingredient of the drug. In this study, the prescribed medications were assigned an antihypertensive drug class based on level 1 ingredient categories associated with ‘Cardiovascular agents’ and level 2 ingredient categories as outlined in a previously published paper [21]. Participants were classified as RHTN if their BP ≥140/90 mmHg and treated with ≥3 classes of antihypertensive medication or BP <140/90 mmHg but treated with ≥4 classes of antihypertensive medication.

### Measurement of blood Pb, Cd, and Hg

The concentrations of Pb, Cd, and Hg in whole blood samples from all eligible participants were determined using inductively coupled-plasma dynamic reaction cell-mass spectrometry (ICP-DRC-MS) at the Division of Laboratory Sciences, National Center for Environmental Health, Atlanta, Georgia. The laboratory details about the methods and quality control/quality assurance data are available on the NHANES website [22]. The limits of detection (LOD) for Pb, Cd, and Hg varied across different NHANES cycles that are summarized in Table S2. The detection rates for Pb, Cd, and Hg were 99.70%, 83.40%, and 92.63%, respectively. Values below the LOD were replaced by the LOD divided by the square root of two according to NHANES analysis guidelines. As all heavy metal measures exhibited skewed distributions, natural log-transformation was applied for statistical analysis. Previous studies have reported that relative to that in urine, heavy metals in blood could be more stable and valid to investigate the associations between metal exposure and a health condition such as HTN [19, 20].

### Covariates

Demographic covariates included age (in years), sex (male and female), race/ethnicity (Mexican American, Other Hispanic, Non-Hispanic White, Non-Hispanic Black, and other), educational level (high school less vs. more than high school), marital status (married or living with partner, and widowed/divorced/separated/never married), annual household income (<$20,000 and ≥$20,000). Other covariates were body mass index (BMI; <18.5,18.5–24.9, 25–29.9, and ≥30), smoking behavior (if smoked at least 100 cigarettes in life), alcohol drinking behavior (if had at least 12 alcohol drinks in the past year), chronic kidney disease (yes/no), diabetes (yes/no). Estimated glomerular filtration rate (eGFR) was estimated using serum creatinine measurement. CKD was defined as eGFR <60 ml/min per 1.73 m^2^ or self-reported kidney disease. Diabetes was defined as Glycohemoglobin ≥6.5% or reported that they had ever been told by a doctor or health professional that you have diabetes. Some of these covariates have been commonly employed in previous analysis of NHANES data [23].

### Statistical analysis

The statistical analysis was conducted using R version 4.1.3 using programs from the “survey” package to account for the complex design used in the construction of the NHANES survey. Results of descriptive analyses presented as weighted mean and standard deviation (SD) for continuous variable and weighted proportion for categorical variables.

Heavy metals were modeled as continuous (Ln-transformed) and categorical defined by quartiles (Q1, Q2, Q3, and Q4). To examine the association between metal exposure and RHTN, bivariate analyses were first performed using weighted rank sum tests for continuous metal measurements and weighted chi-square tests in quartile. Multivariate models were then used to verify the results from bivariate analysis after controlling for covariates. Given the three outcome categories, namely resistant hypertension (RHTN), non-resistant hypertension (NRHTN), and no hypertension (NHTN), we employed multinomial logistic regression modeling to assess the impact of metals on pairwise comparisons among these groups (i.e., RHTN vs. NRHTN, RHTN vs. NHTN, and NRHTN vs. NHTN). The metal measurements were modeled as continuous and in quartile and tendency was tested by entering the quartile as ordinary variables in the model. The effects were shown as odds ratio (OR) and 95% confidence interval (95%CI).

The interactive and combined effects of the metals were estimated to gain insights into whether these metals cast any mixture effects on RHTN. The multinomial logistic regression models were further constructed including the main effect of each metal and the two-metal interactions with adjustment of covariates. The “gWQS” package was used for the weighted quantile sum (WQS) analysis to examine the associations between the metal mixtures and RHTN. In this model, a WQS index was calculated from weighted sums of individual metal concentrations. The model estimated the overall index effect and the contribution of each metal to the overall index.

Missing data was coded as a missing indicator category for the categorical variables. In addition to the total sample, we furthered our analysis stratified by gender (male vs. female), age (<60 years old and >60) and CKD (yes/no), considering potential differences in the metal exposure and RHTN risk among these population subgroups [9, 24]. Statistical significance was set at *p* < 0.05 (two-tailed).

## Results

### Participant characteristics

Final sample consisted of 1365 participants with RHTN, 10562 without RHTN and 26354 with normal BP. Table 1 summarizes sample statistics. There were significant differences in age, gender/sex, race/ethnicity, education level, marital status, annual household income, BMI, smoking behavior, drinking alcohol behavior, CKD, and diabetes among the RHTN, NRHTN, and NHTN groups. Specifically, compared with participants without RHTN, participants with RHTN tended to be old, male, non-Hispanic Black, less educated, obese, not drinking currently, smoking at least 100 cigarettes in life, and having lower annual household income and greater proportion of CKD and diabetes. Compared with normotensive participants, those with RHTN were more likely to be old, male, non-Hispanic Black, less educated, widowed/divorced/separated/never married, obese, smoking at least 100 cigarettes in life, and having lower annual household income and greater proportion of CKD and diabetes. All these characteristics were statistically different between the normotensives and non-RHTN patients.

**Table 1:** Participant characteristics

**Characteristics**	**Total** **n = 38281**	**RHTN** **n = 1365**	**NRHTN** **n = 10562**	**NHTN** **n = 26354**	***p*-Value**
Age, median (IQR)^§#^*	46(30,63)	72(63,80)	55(65,74)	37(25,50)	<0.01
Gender/Sex, n(%)^§#^*					<0.01
Male	18882(49.3)	764(51.8)	4935(44.4)	13183(49.0)	
Female	19399(50.7)	601(48.2)	5627(55.6)	13171(51.0)	
Race/Ethnicity, n(%)^§#^*					<0.01
Mexican American	7102(18.6)	136(3.5)	1313(4.2)	5653(9.9)	
Other Hispanic	3055(8.0)	100(4.3)	712(3.8)	2243(6.3)	
Non-Hispanic White	16910(44.2)	646(69.7)	5113(72.4)	11151(67.3)	
Non-Hispanic Black	7955(20.8)	419(18.5)	2711(14.2)	4825(9.5)	
Other race	3259(8.5)	64(3.8)	713(5.4)	2482(7.1)	
Educational level, n(%)^§#^*					<0.01
High school or below	17623(50.1)	816(55.9)	5894(47.6)	10913(39.1)	
Above high school	17559(49.9)	548(44.1)	4644(52.4)	12367(60.9)	
Marital status^§^*					<0.01
Married/Living with partner	21131(57.6)	750(53.6)	6151(63.9)	14230(62.6)	
Widowed/Divorced/Separated/Never married	15544(42.4)	606(46.4)	4315(36.1)	10623(37.4)	
Annual household income, n(%)^§#^*					<0.01
<$20,000	8190(22.9)	449(30.9)	2752(16.6)	4989(13.7)	
≥$20,000	27539(77.1)	844(69.1)	7129(83.4)	19566(86.3)	
BMI^§#^*					<0.01
<18.5	732(1.9)	6(0.1)	70(0.8)	656(2.3)	
18.5–24.9	11687(31.0)	203(15.7)	1817(15.6)	9667(37.3)	
25–29.9	12470(33.1)	407(27.7)	3461(34.0)	8602(33.5)	
>=30	12841(34.0)	649(56.5)	4959(49.6)	7188(26.9)	
Smoked at least 100 cigarettes in life^§#^*					<0.01
Yes	16154(45.3)	751(53.5)	5228(49.5)	10175(43.8)	
No	19543(54.7)	613(46.5)	5322(50.5)	13608(56.2)	
Drinking at least 12 drinks in the past 1 year^#^*					<0.01
Yes	23619(70.2)	790(66.8)	6375(69.5)	16454(77.7)	
No	10026(29.8)	501(33.2)	3604(30.5)	5921(22.3)	
Chronic kidney disease^§#^*					<0.01
Yes	9136(25.9)	914(67.0)	4495(42.6)	3727(16.0)	
No	26116(74.1)	451(33.0)	6056(57.4)	19609(84.0)	
Diabetes^§#^*					<0.01
Yes	6073(15.9)	705(45.4)	3371(28.7)	1997(7.6)	
No	32205(84.1)	660(54.6)	7191(71.3)	24354(92.4)	

BMI: Body Matrix Index; IQR: Interquartile Range; NHTN: no hypertension; NRHTN: non-resistant hypertension; RHTN: resistant hypertension; §: *p* < 0.05 for RHTN vs. NRHTN; #: *p* < 0.05 for RHTN vs. NHTN; *: *p* < 0.05 for NRHTN vs. NHTN

### Associations of blood Pb with RHTN

Table S3 shows results of bivariate analysis. The blood Pb concentration was significant higher in people with RHTN than that in people with NRHTN (1.70 ug/dL vs. 1.58 ug/dL, *p* < 0.05) and with NHTN (1.70 ug/dL vs. 1.16 ug/dL, *p* < 0.05). When analyzed in quartile, the difference was significant for RHTN vs. NRHTN, RHTN vs. NHTN, and NRHTN vs. NHTN (*p* < 0.05 for all).

**Fig. 2:**
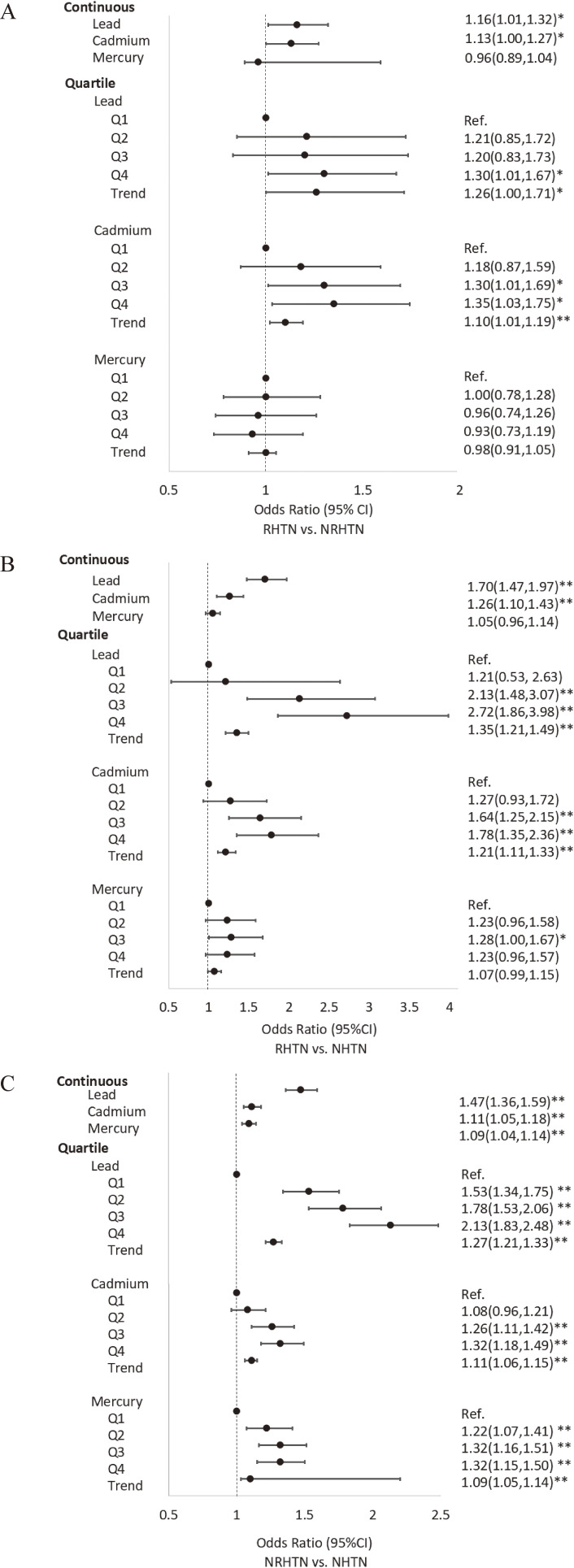
Association of blood lead, cadmium, and mercury with resistant hypertension. CI: confidence interval; RHTN: resistant hypertension; NRHTN: no resistant hypertension; NHTN: no hypertension *p < 0.05; **p < 0.01

After adjusting for covariates, the association between blood Pb concentration and RHTN remained significant. When comparing the RHTN group with the NRHTN group, there was a significant association observed with blood Pb as both a continuous variable (OR[95%] = 1.16[1.01,1.32], *p* < 0.05) and in quartile (OR[95%CI] = 1.30[1.01,1.67] for Q4 vs. Q1, *p* < 0.05) (Fig. 2A). The increasing trend was also significant (OR[95%CI] = 1.26[1.00,1.17], *p* for trend <0.05) (Fig. 2A). Comparing RHTN with NHTN revealed significant association with blood Pb concentration as both a continuous variable (OR[95%] = 1.70[1.47,1.97], *p* < 0.05), as well as in quartile (OR[95%CI] = 2.13[1.48, 3.07] for Q3 vs. Q1; 2.72[1.86,3.98] for Q4 vs. Q1; *p* for trend <0.01) (Fig. 2B). For NRHTN compared to NHTN, the OR[95%CI] was 1.47[1.36,1.59] when analyzing Pb concentration as a continuous variable and 1.53[1.34,1.75] for Q2 vs. Q1, 1.78[1.53,2.06] for Q3 vs. Q1, and 2.13[1.83,2.48] for Q4 vs. Q1 when analyzing Pb concentration in quartile (Fig. 2C).

### Associations of blood Cd with RHTN

Bivariate analysis indicated that RHTN participants had significantly higher Cd concentration than NRHTN participants (0.44 ug/L vs. 0.40 ug/L, *p* < 0.05) and NHTN participants (0.44 ug/L vs. 0.31 ug/L, *p* < 0.05). The differences existed when analyzed by quartiles (*p* < 0.05 for all) (Table S3).

After adjusting for covariates, a significant association was observed between blood Cd concentration as a continuous variable and the RHTN group compared to the NRHTN group (OR[95%CI] = 1.13[1.00,1.27], *p* < 0.05) (Fig. 2A). This association remained significant when analyzing in quartile (OR[95%CI] = 1.30[1.01,1.69] for Q3 vs. Q1 and 1.35[1.03,1.75] for Q4 vs. Q1, *p* for trend <0.05) (Fig. 2A). Similarly, there was a significant association for RHTN vs. NHTN groups with blood Cd concentration as a continuous variable (OR[95%CI] = 1.26[1.10,1.43], *p* < 0.01) and in quartile (OR[95%CI] = 1.64[1.25,2.15] for Q3 vs. Q1 and 1.78[1.35,2.36] for Q4 vs. Q1, p for trend <0.05) (Fig. 2B). When comparing RHTN with NHTN groups, the OR[95%CI] was 1.11[1.05,1.18] for blood Cd concentration as a continuous variable and 1.26[1.11,1.42] for Q3 vs. Q1 and 1.32[1.18,1.49] for Q4 vs. Q1 for blood Cd concentration in quartile (Fig. 2C).

### Associations of blood Hg with RHTN

Bivariate analysis indicated lower blood Hg for RHTN participants than for NHTN participants (0.78 ug/L vs. 0.81 ug/L, *p* < 0.05) and higher blood Hg for NRHTN than for NHTH participants (0.90 ug/L vs. 0.81 ug/L, *p* < 0.05). Similar results were observed when data were analyzed by quartile (Table S3).

After controlling for covariates, multinomial logistic regression confirmed the association of blood Hg with NRHTN relative to NHTN as a continuous variable (OR[95%CI] = 1.09[1.04,1.14], *p* < 0.01) and in quartile (OR[95%CI] = 1.22[1.07,2.41] for Q2 vs. Q1, 1.32[1.16,1.51] for Q3 vs. Q1, and 1.32[1.15,2.50] for Q4 vs. Q1, *p* for trend <0.01) (Fig. 2C).

### Associations of metal mixture with RHTN

There were no interactive effects of any two metals on RHTN (Table S4). Table 2 shows the association of the WQS index and RHTN, as well as the weight of each metal to the WQS index. The WQS index of the blood metal mixture was significantly associated with RHTN. The coefficient and standard error (beta ± SE) for RHTN vs. NRHTN was 0.15 ± 0.04 (*p* < 0.01) and the highest weighted metal was Pb (0.64), followed by Cd (0.25) and Hg (0.11). For RHTN vs. NHTN, the association was statistically significant (0.81 ± 0.04, *p* < 0.01), with Pb as the highest weight (0.59), followed by Cd (0.31) and Hg (0.10). Similarly, the association, for NRHTN vs. NHTN, was also significant (0.52 ± 0.02, *p* < 0.01) and Pb account for the highest weight (0.70), following by Cd (0.25) and Hg (0.05).

**Table 2:** Results of weighted quantile sum analysis

	**Coefficient ± SE**	**p-value**	**weight**
RHTN vs. NRHTN	0.15 ± 0.04	<0.01	-
Lead	-	-	0.64
Cadmium	-	-	0.25
Mercury	-	-	0.11
RHTN vs. NHTN	0.81 ± 0.04	<0.01	-
Lead	-	-	0.59
Cadmium	-	-	0.31
Mercury	-	-	0.10
NRHTN vs. NHTN	0.52 ± 0.02	<0.01	-
Lead	-	-	0.70
Cadmium	-	-	0.25
Mercury	-	-	0.05

NHTN: no hypertension; NRHTN: non-resistant hypertension; RHTN: resistant hypertension; SE: standard error

### Stratification analysis

Male participants had higher levels of blood Pb (1.59 ug/L vs. 1.08 ug/L, *p* < 0.01) and Cd (0.41 ug/L vs. 0.31 ug/L, *p* < 0.01) than female participants (Table 3); people ≤60 years old had higher level of blood Pb (1.75 ug/L vs. 1.1 ug/L, *p* < 0.01), Cd (0.41 ug/L vs. 0.30 ug/L, *p* < 0.01), and Hg (0.89 ug/L vs. 0.81 ug/L, *p* < 0.01) than those who were >60 years old (Table 4); people with CKD had higher level of blood Pb (1.76 ug/L vs. 1.20 ug/L, *p* < 0.01) and Cd (0.42 ug/L vs. 0.35 ug/L, *p* < 0.01) than people had normal kidney function (Table 5).

**Table 3:** Associations of blood lead, Cadmium, and mercury with RHTN stratified by gender

**Heavy metals**	**Metal Concentration** **Median (IQR)**	**RHTN vs. NRHTN** **OR(95%CI)**	**RHTN vs. NHTN** **OR(95%CI)**	**NRHTN vs. NHTN** **OR(95%CI)**
**Male** (n = 18882)				
Lead				
Continuous	1.59(1.03,2.53)	1.18(0.95,1.46)	1.57(1.24,1.99)**	1.33(1.21,1.45)**
By quartile				
Q1	0.62(0.51,0.72)	Ref.	Ref.	Ref.
Q2	1.04(0.93,1.18)	1.17(0.66,2.09)	1.70(0.92,3.75)	1.45(1.19,1.77)**
Q3	1.63(1.48,1.83)	1.09(0.62,1.93)	1.75(0.94,3.25)	1.60(1.32,1.95)**
Q4	3.02(2.47,4.12)	1.33(1.01,2.29)*	2.45(1.29,4.65)**	1.93(1.58,2.35)**
Trend		1.17(1.00,1.24)*	1.35(1.21,1.49)**	1.27(1.21,1.33)**
Cadmium				
Continuous	0.41(0.25,0.62)	1.17(1.01,1.37)	1.21(1.02,1.43)*	1.04(0.96,1.12)
By quartile				
Q1	0.12(0.14,0.22)	Ref.	Ref.	Ref.
Q2	0.30(0.25,0.32)	1.11(0.80,1.56)	1.15(0.83,1.61)	1.03(0.88,1.22)
Q3	0.45(0.42,0.52)	1.28(0.96,1.71)	1.48(1.08,2.02)**	1.15(0.97,1.37)
Q4	1.00(0.77,1.42)	1.39(1.00,1.94)*	1.52(1.35,2.36)**	1.09(0.93,1.29)
Trend		1.12(1.01,1.24)*	1.16(1.04,1.30)*	1.04(0.98,1.09)
Mercury				
Continuous	0.83(0.43,1.73)	0.94(0.83,1.06)	1.01(0.88,1.15)	1.07(0.99,1.16)
By quartile				
Q1	0.23(0.20,0.36)	Ref.	Ref.	Ref.
Q2	0.62(0.52,0.72)	1.11(0.79,1.58)	1.38(0.96,1.99)	1.23(1.03,1.46)**
Q3	1.17(0.99,1.39)	1.02(0.68,1.51)	1.30(0.85,1.99)	1.27(1.06,1.51)**
Q4	3.00(2.18,4.76)	0.92(0.65,1.32)	1.14(0.78,1.68)	1.23(0.99,1.51)
Trend		0.97(0.87,1.07)	1.03(0.92,1.15)	1.06(0.99,1.14)**
**Female** (n = 19399)				
Lead				
Continuous	1.08(0.69,1.70)	1.12(0.94,1.34)	1.81(1.49,2.19)**	1.61(1.45,1.79)**
By quartile				
Q1	0.56(0.43,0.69)	Ref.	Ref.	Ref.
Q2	1.01(0.90,1.15)	1.23(0.82,1.85)	1.83(1.21,2.78)**	1.49(1.26,1.75)**
Q3	1.60(1.41,1.80)	1.32(0.84,2.01)	2.36(1.54,3.62)**	1.79(1.50,2.13)**
Q4	2.79(2.36,3.60)	1.23(0.79,1.91)	2.78(1.71,4.52)**	2.26(1.85,2.77)**
Trend		1.06(0.94,1.20)	1.38(1.21,1.58)**	1.31(1.23,1.39)**
Cadmium				
Continuous	0.31(0.20,0.60)	1.09(0.91,1.31)	1.33(1.08,1.63)**	1.21(1.11,1.32)**
By quartile				
Q1	0.14(0.14,0.20)	Ref.	Ref.	Ref.
Q2	0.28(0.24,0.30)	1.18(0.76,1.82)	1.40(0.89,2.22)	1.19(1.02,1.38)*
Q3	0.44(0.40,0.50)	1.30(0.87,1.95)	1.84(1.22,2.83)**	1.43(1.20,1.70)**
Q4	0.91(0.70,1.34)	1.29(0.84,1.99)	2.12(1.31,2.43)**	1.63(1.37,1.97)**
Trend		1.08(0.96,1.22)	1.28(1.11,1.47)**	1.18(1.11,1.26)**
Mercury				
Continuous	0.84(0.44,1.66)	1.00(0.87,1.14)	1.09(0.95,1.26)	1.10(1.03,1.18)**
By quartile				
Q1	0.23(0.20,0.36)	Ref.	Ref.	Ref.
Q2	0.61(0.52,0.72)	0.89(0.64,1.24)	1.08(0.79,1.47)	1.22(1.01,1.47)*
Q3	1.16(0.99,1.39)	0.93(0.66,1.31)	1.27(0.91,1.77)	1.37(1.15,1.63)**
Q4	2.79(2.10,4.28)	0.97(0.65,1.45)	1.31(0.86,2.02)	1.37(1.13,1.65)**
Trend		1.00(0.88,1.13)	1.10(0.96,1.27)	1.11(1.05,1.18)**

OR: odds ratio; CI: confidence interval; IQR: interquartile range; NHTN: no hypertension; NRHTN: non-resistant hypertension; RHTN: resistant hypertension; *: *p* < 0.05; **p < 0.01.

**Table 4:** Associations of blood lead, cadmium, and mercury with RHTN stratified by age

**Heavy metals**	**Metal Concentration** **Median (IQR)**	**RHTN vs. NRHTN** **OR(95%CI)**	**RHTN vs. NHTN** **OR(95%CI)**	**NRHTN vs. NHTN** **OR(95%CI)**
**≤60 years** (n = 26943)				
Lead				
Continuous	1.75(1.17,2.67)	1.33(1.02,1.75)*	2.24(1.70,2.97)**	1.68(1.53,1.85)**
By quartile				
Q1	0.64(0.52,0.73)	Ref.	Ref.	Ref.
Q2	1.06(0.93,1.18)	1.18(0.88,1.96)	2.18(1.25,3.80)**	1.85(1.56,2.18)**
Q3	1.63(1.46,1.83)	1.31(0.89,2.29)	2.85(1.46,5.57)**	2.18(1.82,2.61)**
Q4	3.00(2.45,4.00)	1.46(1.02,2.68)*	4.02(2.04,7.94)**	2.77(2.30,3.33)**
Trend		1.12(1.00,1.37)*	1.55(1.26,1.90)**	1.38(1.30,1.47)**
Cadmium				
Continuous	0.41(0.29,0.63)	1.20(0.95,1.53)	1.35(1.05,1.74)*	1.12(1.04,1.21)**
By quartile				
Q1	0.14(0.14,0.20)	Ref.	Ref.	Ref.
Q2	0.29(0.25,0.31)	1.06(0.59,1.93)	1.25(0.70,2.25)	1.18(1.02,1.35)*
Q3	0.45(0.40,0.50)	1.33(0.72,2.45)	1.79(0.98,3.28)	1.35(1.16,1.47)**
Q4	0.85(0.70,1.17)	1.76(1.03,3.20)**	2.32(1.28,4.20)**	1.32(1.11,1.55)**
Trend		1.21(1.00,1.47)*	1.34(1.10,1.62)**	1.10(1.05,1.17)**
Mercury				
Continuous	0.89(0.46,1.80)	0.98(0.83,1.17)	1.16(0.97,1.39)	1.17(1.10,1.24)**
By quartile				
Q1	0.23(0.20,0.36)	Ref.	Ref.	Ref.
Q2	0.61(0.52,0.71)	0.55(0.32,0.94)*	0.76(0.46,1.27)	1.39(1.16,1.66)**
Q3	1.16(1.00,1.40)	0.92(0.51,1.65)	1.41(0.79,2.55)	1.51(1.29,1.76)**
Q4	2.80(2.11,4.40)	0.84(0.48,1.45)	1.38(0.79,2.42)	1.60(1.35,1.90)**
Trend		1.00(0.84,1.20)	1.17(0.97,1.42)	1.17(1.10,1.22)
**>60 years** (n = 11338)				
Lead				
Continuous	1.10(0.70,1.80)	1.13(0.98,1.30)	1.11(0.95,1.29)	0.99(0.88,1.10)
By quartile				
Q1	0.57(0.44,0.69)	Ref.	Ref.	Ref.
Q2	1.00(0.90,1.14)	1.30(0.86,1.98)	0.98(0.61,1.56)	0.75(0.57,0.98)*
Q3	1.60(1.41,1.80)	1.24(0.82,1.87)	0.99(0.63,1.55)	0.80(0.62,1.02)
Q4	2.84(2.40,3.80)	1.33(1.10,1.97)*	1.15(0.75,1.79)	0.87(0.67,1.12)
Trend		1.06(0.97,1.17)	1.06(0.96,1.18)	1.00(0.94,1.07)
Cadmium				
Continuous	0.30(0.20,0.58)	1.08(0.95,1.22)	1.12(0.98,1.29)	1.04(0.94,1.16)
By quartile				
Q1	0.14(0.12,0.20)	Ref.	Ref.	Ref.
Q2	0.28(0.23,0.30)	1.19(0.87,1.59)	1.07(0.76,1.52)	0.90(0.73,1.12)
Q3	0.43(0.40,0.50)	1.26(1.01,1.69)	1.32(0.99,1.76)	1.05(0.86,1.28)
Q4	1.00(0.77,1.50)	1.19(1.03,1.75)	1.38(1.03,1.86)*	1.17(0.93,1.46)
Trend		1.05(0.96,1.14)	1.13(1.03,1.24)*	1.08(1.01,1.15)*
Mercury				
Continuous	0.81(0.43,1.64)	0.96(0.87,1.06)	0.91(0.82,1.02)	0.95(0.87,1.03)**
By quartile				
Q1	0.24(0.20,0.36)	Ref.	Ref.	Ref.
Q2	0.61(0.52,0.72)	1.20(0.91,1.59)	1.23(0.94,1.60)	1.01(0.82,1.24)
Q3	1.16(0.98,1.38)	0.98(0.72,1.32)	1.01(0.74,1.39)	1.04(0.82,1.31)
Q4	2.90(2.14,4.56)	0.99(0.73,1.34)	0.90(0.63,1.19)	0.91(0.73,1.12)
Trend		0.97(0.89,1.07)	0.94(0.86,1.03)	0.97(0.91,1.04)

OR: odds ratio; CI: confidence interval; NHTN: no hypertension; NRHTN: non-resistant hypertension; RHTN: resistant hypertension; *: *p* < 0.05; **p < 0.01.

**Table 5:** Associations of blood lead, cadmium, and mercury with resistant hypertension stratified by kidney dysfunction

**Heavy metals**		**RHTN vs. NRHTN** **OR(95%CI)**	**RHTN vs. NHTN** **OR(95%CI)**	**NRHTN vs. NHTN** **OR(95%CI)**
**Kidney dysfunction**				
Lead				
Continuous	1.76(1.14,2.70)	1.28(1.08,1.50)**	1.74(1.43,2.11)**	1.36(1.19,1.56)**
By quartile				
Q1	0.61(0.50,0.71)	Ref.	Ref.	Ref.
Q2	1.04(0.92,1.17)	1.60(0.94,2.71)	2.15(1.20,3.69)	1.32(1.00,1.73)*
Q3	1.62(1.45,1.83)	1.63(0.95,2.79)	2.31(1.32,4.03)*	1.41(1.07,1.88)*
Q4	3.03(2.47,4.10)	1.70(1.03,2.83)*	2.93(1.66,5.16)**	1.72(1.29,2.29)**
Trend		1.12(1.01,1.25)**	1.32(1.15,1.51)**	1.18(1.08,1.28)**
Cadmium				
Continuous	0.42(0.26,0.66)	1.47(1.25,1.72)**	1.22(1.05,1.42)**	0.83(0.76,0.91)**
By quartile				
Q1	0.16(0.15,0.21)	Ref.	Ref.	Ref.
Q2	0.30(0.26,0.32)	1.21(0.86,1.71)	1.34(0.94,1.90)	1.10(0.88,1.39)
Q3	0.47(0.42,0.52)	1.44(1.08,1.93)*	1.76(1.28,2.42)**	1.22(0.98,1.52)
Q4	0.92(0.72,1.32)	1.56(1.15,2.11)**	2.28(1.63,3.21)**	1.47(1.18,1.83)**
Trend		1.16(1.05,1.27)*	1.131(1.17,1.47)**	1.13(1.06,1.21)**
Mercury				
Continuous	0.90(0.47,1.84)	0.97(0.88,1.08)	0.98(0.85,1.07)	0.95(0.89,1.09)
By quartile				
Q1	0.23(0.20,0.36)	Ref.	Ref.	Ref.
Q2	0.62(0.52,0.72)	1.12(0.82,1.54)	1.50(1.11,2.02)**	1.34(1.05,1.71)*
Q3	1.16(0.99,1.39)	0.93(0.65,1.31)	1.00(0.70,1.42)	1.08(0.86,1.36)
Q4	2.86(2.13,4.44)	0.95(0.69,1.30)	0.98(0.70,1.36)	1.03(0.79,1.35)
Trend		0.96(0.88,1.06)	0.95(0.86,1.04)	0.98(0.90,1.07)
**No kidney dysfunction**				
Lead				
Continuous	1.20(0.78,1.91)	0.99(0.78,1.25)	1.48(1.17,1.88)**	1.50(1.37,1.63)**
By quartile				
Q1	0.58(0.45,0.70)	Ref.	Ref.	Ref.
Q2	1.01(0.90,1.15)	0.95(0.62,1.47)	1.49(0.98,2.28)	1.57(1.35,1.83)**
Q3	1.60(1.43,1.80)	0.92(0.59,1.44)	1.72(1.09,2.70)*	1.87(1.59,2.18)**
Q4	2.84(2.40,3.80)	0.98(0.62,1.57)	2.19(1.36,3.53)**	2.23(1.88,2.63)**
Trend		0.99(0.86,1.15)	1.29(1.10,1.49)**	1.28(1.22,1.37)**
Cadmium				
Continuous	0.35(0.20,0.60)	0.99(0.82,1.19)	1.07(0.88,1.30)	1.08(1.02,1.16)*
By quartile				
Q1	0.14(0.13,0.20)	Ref.	Ref.	Ref.
Q2	0.28(0.24,0.30)	1.10(0.70,1.70)	1.18(0.75,1.86)	1.08(0.94,1.24)
Q3	0.44(0.40,0.50)	1.15(1.01,1.72)*	1.46(1.00,2.24)*	1.29(1.12,1.48)**
Q4	0.96(0.73,1.40)	1.04(0.65,1.65)	1.32(0.82,2.11)	1.27(1.10,1.47)**
Trend		1.01(0.88,1.16)	1.11(0.96,1.27)	1.09(1.04,1.15)**
Mercury				
Continuous	0.86(0.45,1.72)	0.96(0.85,1.09)	1.10(0.97,1.25)	1.14(1.07,1.20)**
By quartile				
Q1	0.25(0.20,0.37)	Ref.	Ref.	Ref.
Q2	0.61(0.52,0.72)	0.78(0.55,1.10)	0.94(0.65,1.35)	1.20(1.02,1.40)*
Q3	1.16(0.99,1.39)	1.06(0.72,1.56)	1.55(1.04,2.31)*	1.44(1.23,1.69)**
Q4	2.90(2.15,4.60)	0.94(0.65,1.35)	1.37(0.93,2.01)	1.44(1.24,1.69)**
Trend		1.01(0.91,1.14)	1.16(1.02,1.31)*	1.14(1.08,1.19)**

OR: odds ratio; CI: confidence interval; NHTN: no hypertension; NRHTN: non-resistant hypertension; RHTN: resistant hypertension; *: *p* < 0.05; **p < 0.01.

Tables 3 shows results of the stratified analysis by gender (male/female). The association of blood Pb and Cd with RHTN was significant in male subjects, but not in female. Comparing RHTN with NRHTN among male participants, blood Pb was significantly higher in Q4 vs. Q1 (OR[95%] = 1.33[1.01,2.29], *p* < 0.05) and trend analysis showed a significant dose-response relationship (*p* < 0.05); the association with blood Cd was also significant in Q4 vs. Q1 (OR[95%] = 1.39[1.00,1.94], *p* < 0.05), with a significant result of trend analysis (*p* < 0.05). However, no significant associations were observed among females (Table 3).

After stratified by age, the association of blood Pb and Cd appeared stronger among people ≤60 years than among >60 years old. Blood Pb and Cd, whether as continuous or in quartile, were significant associated with RHTN among people ≤60 years old while the relationship was only significant in Q4 vs. Q1 of blood Cd among people >60 years old (Table 4). The prevalence of CKD was higher among people aged >60 years than those aged ≤60 years (48.60% vs. 15.10%, *p* < 0.01).

Analysis stratified by CKD indicated a stronger association among people with CKD than those without (Table 5). Among people with CKD, blood Pb was significantly associated with RHTN as continuous (OR[95%] = 1.28[1.08,1.50], *p* < 0.01) and in quartile (OR[95%CI] = 1.70[1.03,2.83] for Q4 vs. Q1, *p* for trend <0.01). The association was also significant for blood Cd as a continuous variable (OR[95%] = 1.47[1.25,1.72], *p* < 0.01) and as a quartered variable (OR[95%] = 1.44[1.08,1.93] for Q3 vs. Q1 and 1.56[1.15,2.11] for Q4 vs. Q1, *p* for trend <0.05). Among people without CKD, RHTN was only associated with blood Cd in the Q3 vs. Q1 (OR[95%] = 1.15[1.01,1.72] *p* < 0.05).

We further conducted additional analyses to validate our findings using the diagnostic threshold of 130/80 to define HTN. There was a weaker but still significant relationship between blood Pb and Cd and RHTN (Table S5–S7).

## Discussion

Little is known about the relationship between the environmental metal exposure and RHTN. Our study examined associations of blood Pb, Cd, and Hg with RHTN among non-institutionalized adults in NHANES database. After controlling for potential confounders, we found a relationship between individual blood Pb and Cd and the mixture of Pb, Hg, and Cd in blood and elevated prevalence of RHTN, with blood Pb and Cd accounting for the highest weight. We also found that the associations were stronger among males, people ≤60 years old, and people with CKD.

Compared with normotensive individuals, blood Pb, Cd, and Hg levels were generally associated with increased prevalence of HTN, including RHTN and NRHTN. The finding was consistent with the previous findings that Pb, Cd, and Hg are the top 3 risk factors of metal exposure associated HTN [11]. Beyond present studies, we further found that people with RHTN had higher blood Pb and Cd concentrations than people without RHTN and the WQS analyses showed positive correlations of the mixture of Pb, Hg, and Cd in blood with increased prevalence of RHTN, primarily driven by blood Pb and Cd. Risk factors of RHTN are believed to include medication nonadherence, psychological factors (e.g. white-coat effect), life-style factors (e.g. obesity, high dietary salt, and physical inactivity), use of certain pharmacologic agents (e.g. nonsteroidal anti-inflammatory agents and immunosuppressive agents), and sleep disorders factors (e.g. obstructive sleep apnea) [2, 25]. However, to the best of our knowledge, this is the first study to investigate the relationship between blood concentrations of multiple metals and RHTN.

In the present study, we found that the internal dose of environmental exposure to Pb and Cd may be linked to higher prevalence of RHTN. Because these heavy metals had relatively long biological half-life, they are likely to induce chronic exposure and persistent toxicological effects. The half-life of Pb in adult human blood was estimated to be 30 days but may lead up to 3 years in the brain [26]. Cd can accumulate in the kidney, liver, and bone, with up to 30 years of half-life in the living organisms [26]. Additionally, the half-life of blood mercury was also estimated to be approximately 50 days [27]. Therefore, chronic exposure to these heavy metals due to their long half-life may result in adverse health impacts in a long run. For example, *in vivo* experiments have established that chronic exposure to low levels of Pb can cause arterial HTN that persist long after the cessation of lead exposure [28]. Zheutlin et al. discovered that tibia lead level was linked with elevated RHTN risk in a prospective cohort study [15]. Pb exposure may impact BP through reductions in renal function, oxidative stress, stimulation of the renin-angiotensin system, downregulation of nitric oxide production, and desensitization of β-adrenergic receptors, leading to increased vascular tone and peripheral vascular resistance [28–30]. However, this study only measured blood concentrations of these heavy metal once at the baseline, which may not reflect variations of internal metal dose in a long term. Another reason might be an impact of Pb exposure on the activity of antihypertensive medications. A study found a significant association of blood Pb and uncontrolled BP among all treated hypertensive participants except for those who were taking angiotensin receptor blocker [9]. To the best of our knowledge, we are the first to report that blood Cd concentration was significantly associated with RHTN. Cd exposure is known to induce HTN through endothelial dysfunction, increased peripheral resistance through RAS activation, and nephropathy [31]. These biological mechanisms may be keys for future research directions to better understand the pathogenesis underlying RHTN of Cd exposure.

We found a greater effect size between blood Pb and Cd and RHTN in people ≤60 years old than those >60 years old. The mechanism underlying the age difference was unclear. Heavy metal exposure is more prevalent in young people, and they are more susceptible to occupational exposure to heavy metals. In this study, participants ≤60 years old had higher levels of blood Pb and Cd than those older than 60 years old. In addition, old people may tend to have a higher likelihood of awareness and treatment of HTN due to regular check-ups [32]. We also found a stronger relationship of blood Pb and Cd with RHTN in males than in females. Findings of previous studies showed that the negative cardiovascular effects of exposure to Pb and Cd varied in women and men [9, 16–18]. Environmental and occupational exposures, sex-hormone related differences in metabolism, and storage, excretion and storage of heavy metals may be different between males and females [33, 34]. Exposure to environmental metals may come from various sources such as water, food, and air pollution through different routes of exposure including ingestion, inhalation, and dermal contact. Although not the primary focus of this study, future endeavors should be directed towards investigating the environmental origins of heavy metal exposure in order to develop targeted intervention strategies aimed at reducing both the levels of exposure and associated adverse health effects. Other reasons contribute to the gender- and age-difference include disparate definitions of HTN, heterogenous study samples, and various confounders adjusted for in analysis. Additional studies to explore mechanisms underlying the age and gender difference in the effects of metal exposure on RHTN are merited.

We found the effect of blood metal concentrations on RHTN is obvious in people with CKD. The prevalence of RHTN is elevated in patients with CKD while the prognosis of CKD is much poorer among patients with RHTN than those without [6]. CKD may have already changed the physiological structure of the kidney, leading to an accumulation of metal in the blood [19, 20]. Previous studies have reported that urinary metal concentration decreased with declining renal function whereas the association with blood metals did not change [19, 20]. The excessive metals in body may increase the excretion of metals, which may increase the kidney burden, inducing or exacerbating hypertensive renal diseases. The renal dysfunction may lead to a decrease in the metal excretion rate, resulting in further accumulation of metals in the body, forming a vicious cycle.

This study was conducted using the NHANES datasets consisting of a representative sample of the U.S. population. Blood Pb, Hg, and Cd were analyzed, which provided a thorough evaluation of individual and combined relationship between the metals and RHTN. These findings offer valuable insights for future interventions aimed at reducing the incidence of RHTN by implementing strategies to mitigate heavy metal exposure, such as soil remediation techniques [35]. However, there are several limitations. The results only showed an association between metal exposure and RHTN rather than a causal relationship due to the nature of a cross-sectional study design. In addition, despite controlling for covariates, there are still residual and unmeasured confounders and measurement errors that may bias our results. Moreover, the effects of the residential areas and occupational exposures were not considered in the analysis due to the unavailability of data across all NHANES cycles or lack of public accessibility. Lastly, data on the antihypertensive medication adherence and their doses were unavailable, which limited our understanding of how these factors may impact the metal-RHTN relationship.

In conclusion, we identified that blood Pb and Cd concentrations were associated with increased prevalence of RHTN. The mixture-exposure analyses also showed a positive correlation between a mixture of blood Pb, Cd, and Hg and elevated probability of RHTN, primarily driven by blood Cd and Pb. These findings should warrant future research to better understand the causality and its underlying pathophysiology. Data on details of antihypertensive medication usage should be collected to clarify the impact of medications on this relationship. The results of this study highlighted potential adverse impacts of exposure to environmental metals on BP management among hypertensive patients, particularly for male participants and patients ≤60 years old or with CKD.

## Supplementary information

**Additional file 1:** **Table S1** Characteristics between people included in and excluded from the analysis. **Table S2** The limit of detection for lead, cadmium, and mercury in each NHANES cycle. **Table S3** Comparisons of blood concentrations of lead, cadmium, and mercury between people with RHTN, NRHTN, and NHTN. **Table S4** Interaction between metals on resistant hypertension. **Table S5** Association of each metal with resistant hypertension defined with BP <130/80 from multinomial logistic regression analysis. **Table S6** Interaction between metals on resistant hypertension defined with BP <130/80. **Table S7** The association between WQS index and resistant hypertension defined by BP <130/80 and the weight of each metal to the WQS index.

## Declarations

### Availability of data and materials

NHANES data are openly available in a public repository.
