# Supplementary material for: Associations of blood lead, cadmium, and mercury with resistant hypertension among adults in NHANES, 1999–2018
Source: Environ Health Prev Med. 2023 Nov 2;28:66. doi: 10.1265/ehpm.23-00151 (PMC10636284; doi:10.1265/ehpm.23-00151)
Supplement: Supplementary file 1 — Additional file 1: Table S1 Characteristics between people included in and excluded from the analysis. Table S2 The limit of detection for lead, cadmium, and mercury in each NHANES cycle. Table S3 Comparisons of blood concentrations of lead, cadmium, and mercury between people with RHTN, NRHTN, and NHTN. Table S4 Interaction between metals on resistant hypertension. Table S5 Association of each metal with resistant hypertension defined with BP <130/80 from multinomial logistic regression analysis. Table S6 Interaction between metals on resistant hypertension defined with BP <130/80. Table S7 The association between WQS index and resistant hypertension defined by BP <130/80 and the weight of each metal to the WQS index. [file ehpm-28-066-s001.docx]

Table S1 Characteristics between people included in and excluded from the analysis.

|  | Included  n=38281 | Excluded | | | |
| --- | --- | --- | --- | --- | --- |
|  |  | BP$\geq$140/90 but not using medication  n=8310 | *p*-value | Missing data on BP and metal measurements  n=10941 | *p*-value |
| Age, median (IQR) | 46(30,63) | 53(39,63) | 0.03 | 48(31,66) | 0.23 |
| Gender, n(%) |  |  | <0.01 |  | 0.03 |
| Male | 18882(48.0) | 4510(55.5) |  | 5157 (39.8) |  |
| Female | 19399(52.0) | 3808(44.5) |  | 5784(60.2) |  |
| Race, n(%) |  |  | <0.01 |  | <0.01 |
| Mexican American | 7102(8.4) | 1485(7.47) |  | 1786(9.0) |  |
| Other Hispanic | 3055(5.6) | 655(4.98) |  | 988(8.8) |  |
| Non-Hispanic White | 16910(68.6) | 3552(68.60) |  | 4266(56.2) |  |
| Non-Hispanic Black | 7955(10.9) | 1935(12.47) |  | 2483(15.3) |  |
| Other race | 3259(6.6) | 691(6.47) |  | 1418(10.7) |  |
| Educational level, n(%) |  |  | <0.01 |  | <0.01 |
| High school or below | 17623(41.6) | 4403(46.1) |  | 5129(50.8) |  |
| Above high school | 17559(58.4) | 3667(53.9) |  | 5041(49.2) |  |
| Marital status |  |  | 0.30 |  | 0.06 |
| Married/Living with partner | 21131(62.7) | 4653(63.5) |  | 5672(58.9) |  |
| Widowed/Divorced/Separated/Never married | 15544(37.3) | 3474(36.5) |  | 4693(41.1) |  |
| Household income, n(%) |  |  | <0.01 |  | <0.01 |
| <$20,000 | 8190(15.4) | 1996(17.7) |  | 1475(20.9) |  |
| ≥$20,000 | 27539(84.6) | 5719(82.3) |  | 7342(79.1) |  |
| BMI |  |  | <0.01 |  | 0.5 |
| <18.5 | 732(1.9) | 123(1.58) |  | 175 (1.2) |  |
| 18.5-24.9 | 11687(31.7) | 1947(22.38) |  | 2385 (32.8) |  |
| 25-29.9 | 12470(33.5) | 2702(33.44) |  | 2521(32.6) |  |
| >=30 | 12841(33.0) | 3183(42.60) |  | 2880(33.3) |  |
| Smoked at least 100 cigarettes in life |  |  | <0.01 |  | 0.06 |
| Yes | 10175(45.5) | 4064(52.6) |  | 3519(43.8) |  |
| No | 13608(54.5) | 4055(47.4) |  | 3311(56.2) |  |
| Drinking at least 12 drinks in the past 1 year |  |  | 0.8 |  | 0.03 |
| Yes | 23619(75.4) | 5192(75.2) |  | 4199(69.4) |  |
| No | 10026 (24.6) | 2137(24.8) |  | 2204(30.6) |  |
| Chronic kidney disease |  |  | <0.01 |  | <0.01 |
| Yes | 9136(16.0) | 2453(26.1) |  | 4495(42.6) |  |
| No | 19609(84.0) | 5638(73.9) |  | 6056(57.4) |  |
| Diabetes |  |  | 0.7 |  | 0.02 |
| Yes | 6073(23.3) | 1625(26.9) |  | 5495(27.0) |  |
| No | 16116(76.6) | 6692(73.1) |  | 4714(73.0) |  |

BMI: Body Mass Index; IQR: Interquartile Range

Table S2 The limit of detection for lead, cadmium, and mercury in each NHANES cycle

| NHANES cycle | lead, ug/dL | cadmium, ug/L | mercury, ug/L |
| --- | --- | --- | --- |
| 2017-2018 | 0.07 | 0.10 | 0.28 |
| 2015-2016 | 0.07 | 0.10 | 0.28 |
| 2013-2014 | 0.07 | 0.10 | 0.28 |
| 2011-2012 | 0.25 | 0.16 | 0.16 |
| 2009-2010 | 0.25 | 0.20 | 0.33 |
| 2007-2008 | 0.25 | 0.20 | 0.28 |
| 2005-2006 | 0.25 | 0.20 | 0.20 |
| 2003-2004 | 0.28 | 0.14 | 0.14 |
| 2001-2002 | 0.28 | 0.28 | 0.10 |
| 1999-2000 | 0.28 | 0.28 | 0.10 |

Table S3. Comparisons of blood concentrations of lead, cadmium, and mercury between people with RHTN, NRHTN, and NHTN

| Heavy metal | Total  n=38281 | RHTN  n=1365 | NRHTN  n=10562 | NHTN  n=26354 | *p*-value |
| --- | --- | --- | --- | --- | --- |
| Lead |  |  |  |  |  |
| Level, ug/dL^§#*^ |  |  |  |  | <0.01 |
| median(IRQ) | 1.30(0.80,2.09) | 1.70(1.16,2.69) | 1.58 (1.01, 2.41) | 1.16 (0.71, 1.90) |  |
| Quartile(Q), n(%)^§#*^ |  |  |  |  | <0.01 |
| Q1: | 9431 | 129(12.5) | 1490(17.8) | 7812(33.6) |  |
| Q2: | 9430 | 319(24.2) | 2449(26.0) | 6662(25.9) |  |
| Q3: | 9430 | 381(33.7) | 2996(28.2) | 6053(22.2) |  |
| Q4: | 9430 | 511(29.6) | 3472(28.0) | 5447(18.4) |  |
| Cadmium |  |  |  |  |  |
| Level, ug/L^§#*^ |  |  |  |  | <0.01 |
| median(IRQ) | 0.35(0.20,0.60) | 0.44(0.27,0.65) | 0.40(0.25,0.64) | 0.31(0.20,0.58) |  |
| Quartile(Q), n(%)^§#*^ |  |  |  |  | <0.01 |
| Q1: | 9431 | 180(15.7) | 1785(21.1) | 7466(31.9) |  |
| Q2: | 9430 | 341(23.9) | 2523(25.1) | 6566(26.0) |  |
| Q3: | 9430 | 424(33.5) | 3141(28.0) | 5865(20.2) |  |
| Q4: | 9430 | 395(26.9) | 2958(25.8) | 6077(21.9) |  |
| Mercury |  |  |  |  |  |
| Level, ug/L^#*^ |  |  |  |  | 0.02 |
| median(IRQ) | 0.83(0.44,1.70) | 0.78(0.44,1.54) | 0.90(0.47,1.79) | 0.81(0.42,1.66) |  |
| Quartile(Q), n(%)^#*^ |  |  |  |  | <0.01 |
| Q1: | 8100 | 279(19.6) | 1983(22.7) | 5835(26.0) |  |
| Q2: | 8099 | 339(26.8) | 2164(25.2) | 5596(23.6) |  |
| Q3: | 8099 | 281(30.2) | 2276(26.8) | 5542(24.7) |  |
| Q4: | 8099 | 260(23.5) | 2327(25.3) | 5512(25.7) |  |

IQR： inter-quarter range; NHTN: no hypertension; NRHTN: non-resistant hypertension; RHTN: resistant hypertension; §: *p*<0.05 for RHTN vs. NRHTN; #: *p*<0.05 for RHTN vs. NHTN; *: *p*<0.05 for NRHTN vs. NHTN

Table S4 Interaction between metals on resistant hypertension

|  | RHTN vs. NRHTN | | RHTN vs. NHTN | | NRHTN vs. NHTN | |
| --- | --- | --- | --- | --- | --- | --- |
|  | OR(95%CI) | p-value | OR(95%CI) | p-value | OR(95%CI) | p-value |
| lead×mercury | 1.04(0.92,1.18) | 0.508 | 0.99(0.87,1.02) | 0.873 | 0.95(0.89,1.02) | 0.135 |
| mercury×cadmium | 1.00(0.89,1.12) | 0.988 | 0.96(0.86,1.08) | 0.499 | 0.96(0.91,1.02) | 0.158 |
| lead×cadmium | 1.01(0.86,1.18) | 0.924 | 1.01(0.86,1.19) | 0.903 | 1.00(0.93,1.08) | 0.946 |

CI: confidence interval; NHTN: no hypertension; NRHTN: non-resistant hypertension; OR: odds ratio; RHTN: resistant hypertension;

Table S5 Association of each metal with resistant hypertension defined with BP<130/80 from multinomial logistic regression analysis

|  | RHTN  n=1719 | | |  | NRHTN  n=10208 | |  | NHTN  n=21061 |
| --- | --- | --- | --- | --- | --- | --- | --- | --- |
|  | n(%)/M(IRQ) | RHTN vs. NRHTN  OR(95%CI) | RHTN vs. NHTN  OR(95%CI) |  | n(%)/M(IRQ) | RHTN vs. NHTN  OR(95%CI) |  | n(%)/M(IRQ) |
| Lead |  |  |  |  |  |  |  |  |
| Continuous | 1.70(1.14, 2.67) | 1.13(1.01,1.29)* | 1.79 (1.56,2.06)** |  | 1.58(1.01,2.40) | 1.58 (1.46,1.71)** |  | 1.10(0.70,1.80) |
| Quartile |  |  |  |  |  |  |  |  |
| Q1 | 174(12.8) | Ref. | Ref. |  | 1408(17.5) | Ref. |  | 6545(35.2) |
| Q2 | 372(22.6) | 1.05(0.78,1.42) | 1.71(1.26,2.32)** |  | 2340(25.8) | 1.63(1.41,1.87) ** |  | 5415(26.1) |
| Q3 | 644(29.8) | 1.14(0.82,1.58) | 2.25(1.63,3.12)** |  | 2879(28.2) | 1.98(1.71,2.30) ** |  | 4745(21.8) |
| Q4 | 502(34.8) | 1.19(1.00,1.53)* | 2.76(1.99,3.84)** |  | 3428(28.5) | 2.36(2.00,2.87) ** |  | 4054(16.9) |
| Trend |  | 1.05(0.99,1.13) | 1.38(1.26,1.52)** |  |  | 1.33(1.27,1.40)** |  |  |
| Cadmium |  |  |  |  |  |  |  |  |
| Continuous | 0.43(0.27,0.66) | 1.11(1.00,1.51)* | 1.35(1.11,1.51)** |  | 0.40(0.25,0.46) | 1.14(1.08,1.22)** |  | 0.30(0.20,0.56) |
| Quartile |  |  |  |  |  |  |  |  |
| Q1 | 244(17.6) | Ref. | Ref. |  | 1724(21.0) | Ref. |  | 6159(33.0) |
| Q2 | 419(23.1) | 1.06(0.81,1.38) | 1.20(0.91,1.59) |  | 2441(25.2) | 1.13(1.01,1.27)* |  | 5267(25.7) |
| Q3 | 533(33.0) | 1.14(0.89,1.45) | 1.53(1.18,1.98)** |  | 3027(27.9) | 1.35(1.17,1.55)** |  | 4566(19.7) |
| Q4 | 496(26.3) | 1.21(1.01,1.59)* | 1.76(1.38,2.25)** |  | 2863(25.9) | 1.41(1.24,1.60)** |  | 4767(21.6) |
| Trend |  | 1.06(0.98,1.14) | 1.19(1.10,1.30)** |  |  | 1.13(1.08,1.18)** |  |  |
| Mercury |  |  |  |  |  |  |  |  |
| Continuous | 0.80(0.45,1.60) | 0.96(0.89,1.03) | 1.04(0.96,1.13) |  | 0.89(0.47,1.79) | 1.09(1.04,1.15)** |  | 0.8(0.42,1.63) |
| Quartile |  |  |  |  |  |  |  |  |
| Q1 | 350(21.9) | Ref. | Ref. |  | 1912(22.5) | Ref. |  | 4794(25.9)- |
| Q2 | 413(26.8) | 0.98(0.80,1.21) | 1.23(0.96,1.55)# |  | 2072(25.0) | 1.25(1.08,1.44) ** |  | 4571(24.3) |
| Q3 | 359(27.6) | 0.94(0.76,1.16) | 1.32(1.03,1.68)* |  | 2202(27.0) | 1.40(1.23,1.60) ** |  | 4494(24.0) |
| Q4 | 341(23.7) | 0.92(0.75,1.14) | 1.21(0.95,1.53) |  | 2260(25.6) | 1.32(1.15,1.51) ** |  | 4454(25.8) |
| Trend |  | 0.97(0.91,1.04) | 1.06(0.99,1.14)# |  |  | 1.10(1.05,1.14)** |  |  |

Table S6 Interaction between metals on resistant hypertension defined with BP<130/80

|  | RHTN vs. NHTN | | RHTN vs. NHTN | | NRHTN vs. NHTN | |
| --- | --- | --- | --- | --- | --- | --- |
|  | OR(95%CI) | p-value | OR(95%CI) | p-value | OR(95%CI) | p-value |
| PB×HG | 1.05(0.93,1.18) | 0.434 | 1.00(0.88,1.13) | 0.173 | 0.95(0.88,1.02) | 0.295 |
| HG×CD | 1.03(0.92,1.15) | 0.622 | 0.99(0.89,1.11) | 0.259 | 1.00(0.91,1.03) | 0.178 |
| PB×CD | 1.01(0.87,1.17) | 0.871 | 1.00(0.86,1.16) | 0.793 | 0.99(0.92,1.07) | 0.763 |

CI: confidence interval; NHTN: no hypertension; NRHTN: non-resistant hypertension; OR: odds ratio; RHTN: resistant hypertension;

Table S7 The association between WQS index and resistant hypertension defined by BP<130/80 and the weight of each metal to the WQS index.

|  | Coef. ± SE | p-value | weight |
| --- | --- | --- | --- |
| RHTN vs. NRHTN | 0.12±0.03 | <0.001 | - |
| Lead | - | - | 0.61 |
| Cadmium | - | - | 0.27 |
| Mercury | - | - | 0.12 |
| RHTN vs. NHTN | 0.77±0.04 | <0.001 | - |
| Lead | - | - | 0.73 |
| Cadmium | - | - | 0.27 |
| Mercury | - | - | 0.00 |
| NRHTN vs. NHTN | 0.54±0.02 | <0.001 | - |
| Lead | - | - | 0.78 |
| Cadmium | - | - | 0.20 |
| Mercury | - | - | 0.01 |

NHTN: no hypertension; NRHTN: non-resistant hypertension; RHTN: resistant hypertension; SE: standard error
